# Current practice patterns and gaps in guideline-concordant breast cancer survivorship care

**DOI:** 10.1007/s11764-021-01152-1

**Published:** 2021-12-30

**Authors:** Eden R. Brauer, Elisa F. Long, Laura Petersen, Patricia A. Ganz

**Affiliations:** 1 School of Nursing, University of California, Los Angeles (UCLA), Los Angeles, CA USA; 2 Anderson School of Management, University of California, Los Angeles (UCLA), Los Angeles, CA USA; 3 Cancer Prevention and Control Research, University of California, Los Angeles (UCLA), Los Angeles, CA USA; 4 David Geffen School of Medicine and Fielding School of Public Health, University of California, Los Angeles (UCLA), Los Angeles, CA USA

**Keywords:** Cancer survivorship, Breast cancer, Long-term and late effects

## Abstract

**Purpose:**

Breast cancer-specific survivorship care guidelines for the more than 3.8 million survivors in the U.S. are available, but implementation in clinical practice remains challenging. We examined current practice patterns and factors associated with guideline-concordant survivorship care among oncologists.

**Methods:**

A national sample of medical oncologists, recruited using two databases, participated in a survey focused on practice patterns for breast cancer survivorship care. A “survivorship care composite score” was calculated for each respondent based on provision of services recommended in the survivorship guidelines. Descriptive statistics and multivariable linear regression analyses examined associations between physician and practice characteristics and composite scores.

**Results:**

The survey was completed by 217 medical oncologists, with an overall response rate of 17.9% and eligibility rate of 56.9% for those who responded. Oncologists reported high engagement in evaluation of disease recurrence (78%). Performed less frequently were the provision of survivorship care plans (46%), assessment of psychosocial long-term and late effects (34%), and screening for subsequent cancers (34%). Lack of survivorship care training (*p* = 0.038) and not routinely informing patients about potential late effects (*p* = 0.003) were significantly associated with poorer survivorship care composite scores.

**Conclusions:**

Despite the availability of disease-specific survivorship care guidelines, adherence to their recommendations in clinical practice is suboptimal. Survey results identified key gaps in survivorship care for breast cancer survivors, particularly related to subsequent primary cancers and psychosocial long-term and late effects.

**Implications for Cancer Survivors:**

Improving the delivery of comprehensive survivorship care for the growing population of breast cancer survivors is a high priority. Disease-specific clinical guidelines for cancer survivorship provide valuable recommendations, but innovative strategies are needed to integrate them into the care of long-term breast cancer survivors.

**Supplementary Information:**

The online version contains supplementary material available at 10.1007/s11764-021-01152-1.

With more than 280,000 breast cancer diagnoses in the United States (U.S.) annually—15 percent of all new cancer cases—and continued improvements in early detection and adjuvant therapies, the number of living breast cancer survivors is expected to increase from 3.8 million in 2020 to nearly 5 million by 2030 [1, 2]. The 5-year female breast cancer survival rate in the U.S. is approximately 90%, and exceeds 98% in women with localized disease at diagnosis [1]. According to Surveillance, Epidemiology and End Results (SEER) program data, the majority of breast cancer survivors (71%) were diagnosed more than 5 years ago, with nearly one in five diagnosed more than 20 years ago [3]. Given that most women with breast cancer have excellent prognoses and will experience long-term survival, access to high-quality, comprehensive survivorship care is a key focus for cancer care delivery research.

Despite advancements in early detection, treatment, and survival, breast cancer survivors often experience health consequences related to their disease and/or treatments well beyond initial diagnosis and treatment. In addition to metastatic cancer recurrence, survivors are at risk for subsequent primary cancers [4], comorbid conditions [5], and a wide range of physical and psychosocial long-term and late effects [6, 7]. These persistent difficulties have social, financial, and practical consequences for survivors, often impairing resumption of normal activities, employment, and social roles. Adding to the complexity is the interplay with aging and other chronic diseases.

Clinical guidelines for cancer survivorship care have been developed by multiple organizations, including the American Cancer Society (ACS), National Comprehensive Cancer Network (NCCN), and American Society of Clinical Oncology (ASCO). Recognizing the need for disease-specific recommendations, ACS and ASCO collaboratively developed survivorship care guidelines for major cancer types, including breast cancer, to facilitate the translation of evidence into clinical practice [8]. These guidelines provide breast cancer-specific information through a framework of the essential domains of survivorship care: (a) surveillance for disease recurrence, (b) subsequent cancer screening, (c) management of long-term and late physical and psychosocial effects, and (d) health promotion and care coordination.

Despite these efforts, serious gaps persist in the comprehensive care of long-term breast cancer survivors [7, 9]. To better understand adherence to breast cancer survivorship care guidelines, we surveyed a sample of medical oncologists to assess current practice patterns and examine factors associated with guideline-concordant survivorship care.

Methods

## Survey development

Informed by our prior qualitative research with medical oncologists and breast cancer survivors [10], we developed a 60-item electronic survey about practice patterns in breast cancer survivorship care, based on the ACS/ASCO guidelines (Appendix 1). Prior to study initiation, the survey was pilot-tested by medical oncologists (*n*=6) regarding content and electronic administration, and amended based on their feedback. The survey began with a core component and then randomized respondents to one of three patient vignettes to assess knowledge of guideline-specific management strategies. In this report, we examine only the survey core component.

### Sample and recruitment

Medical oncologists were eligible for the survey if they were currently treating at least one breast cancer patient in their practice. To recruit a national sample, we obtained a database of approximately 6000 individuals from a commercial vendor (SK&A). Although primarily created for marketing purposes, the SK&A database contains data on health care providers, and has been used extensively in health services research [11–17]. Prior studies suggest that the SK&A database is reasonably accurate and up-to-date, and significantly outperforms the AMA Masterfile, particularly as a source of contact information for physicians [11, 18]. Email invitations were sent to 1500 randomly selected individuals from the SK&A database, with up to five reminders (electronic and postal) to non-respondents. As a second recruitment strategy, we obtained access to the ASCO Center for Research and Analytics survey pool, which includes professional members who have agreed to be contacted for research purposes. When available, reasons for non-participation were recorded; however, due to the recruitment methods, we could not verify that invitation letters were received.

### Survey administration

Medical oncologists were invited to participate in the survey; interested individuals were sent a unique link or hardcopy when preferred. The survey began with a self-administered consent and two eligibility questions to determine if the individual was (1) a medical oncologist, (2) who treats patients with breast cancer, and took approximately 15–20 minutes to complete. Responses were collected and stored in a secure, web-based application (REDCap). Upon survey completion, participants received a $50 gift card.

### Survey content and outcomes

To explore differences in practice patterns of survivorship care, we collected self-reported data on physician and practice characteristics. Physician characteristics included age, gender, race, year of medical school graduation, faculty affiliation with a medical school, weekly time spent in direct patient care, typical patient volume, and receipt of additional training in survivorship care. Practice characteristics included practice type, geographic region, and whether the practice was participating in the Oncology Care Model (OCM), a value-based oncology care model being tested by the Centers for Medicare & Medicaid Services at the time of this study.

Aspects of survivorship care delivery at the practice level were queried, such as usual duration, clinician, and setting for post-treatment appointments. Oncologists were asked whether they routinely inform patients about potential long-term and late effects (yes/no), and if so, the timing of these discussions in a patient’s trajectory (before, during, or after treatment). The use of formal consent when initiating chemotherapy was also assessed, as we hypothesized that informing patients about potential long-term and late effects might occur as part of a broader informed consent discussion on risks and benefits of treatment.

The main outcome variable was routine delivery of breast cancer survivorship care, based on core services described in the current ACS/ASCO guidelines. In this study, core survivorship services included (1) evaluating for disease recurrence, (2) providing formal survivorship care plans, (3) communicating with patients’ other physicians about follow-up care, (4) discussing plans for survivorship care with patients, (5) screening for new primary cancers, (6) assessing and managing adverse physical and (7) psychosocial long-term and late effects, and (8) health promotion and counseling on diet, physical activity, and (9) smoking cessation. Oncologists were asked to report how frequently breast cancer survivors in their practice receive the services, with Likert-type responses from “rarely/never” to “always/almost always.” Responses to the core services were then combined into an overall measure, a survivorship care composite score. A composite score approach was utilized in an effort to represent high-quality, comprehensive survivorship care as a set of essential services rather than a single action (e.g., delivery of survivorship care plan).

### Data analysis

Descriptive statistics of physician and practice characteristics and frequencies of the nine core services were calculated. Associations between physician and practice characteristics and high engagement, defined as “always/almost always,” in the core services were examined using logistic regression. To calculate an overall survivorship care composite score for each participant, responses in the nine core services were numerically coded with higher scores corresponding to greater frequency (5 = “always/almost always” and 1 = “rarely/never”) and then averaged, with the contributions of the component variables considered equal [19, 20].

Relationships between physician and practice characteristics and survivorship care composite scores were analyzed using Student’s *t* tests and ANOVA. Multivariable linear regression analysis was then conducted to examine which physician and practice characteristics were independently associated with the survivorship care composite score. For the final regression model, the composite score was log transformed to correct for data skewness, resulting in a continuous variable. All calculations were performed in R Studio, version 1.3.

The research was reviewed and approved by the UCLA Institutional Review Board.

#### Results

### Recruitment outcomes

**Fig. 1:**
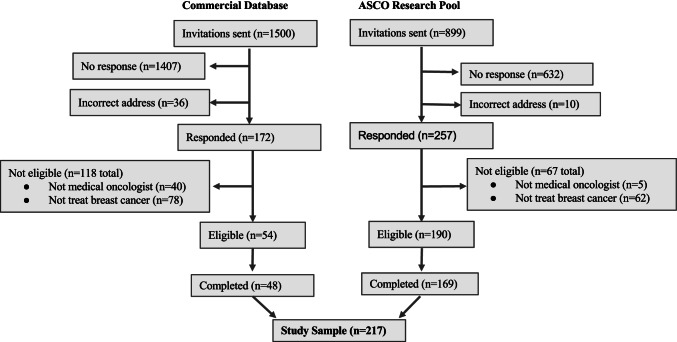
Study recruitment diagram

Between October, 2018 and April, 2020, we attempted to contact a random sample of medical oncologists from the commercial database (*n* = 1500) and subsequently the ASCO pool (*n* = 899) about study participation (Figure 1). A total of 429 individuals responded for an overall response rate of 17.9%, and eligibility rate of 56.9% for those who responded. Forty-three percent were deemed ineligible, primarily because they did not treat any patients with breast cancer (140/429) or did not identify as a medical oncologist (e.g., other specialty) (45/429). Two hundred seventeen completed the survey during the study.

The final sample was mainly recruited through the ASCO pool (*n* = 169/217, 78%). Of the 1500 individuals from the commercial database, 172 individuals (11.5%) responded, and of these, 54 were eligible and 48 completed the survey. Mailed follow-up letters to 500 non-respondents yielded 18 of these surveys. In the ASCO pool, 257/899 individuals (28.6%) responded, and of these, 190 were eligible, and 169 completed the survey. For the ASCO sample, we compared the distribution of respondents and non-respondents by gender, age, ethnicity, region, practice setting, and membership type using Fisher’s exact tests. No significant differences were observed except by membership type, with early career members more likely to respond (42%) than regular members (27%) or members in training (25%) (*p* =.002). Information about non-respondents from the commercial database was not available. When comparing respondents from the two recruitment groups, significant differences were observed with respect to age, with respondents from the commercial database 18 years older on average than ASCO respondents, and time since medical school graduation (*p*<.0001). The ASCO group was also less likely to identify as white (49%) compared to the commercial group (76%) (*p*<.002). No significant differences were observed by gender, medical training in the U.S., having a faculty appointment, or formal training in cancer survivorship between the two recruitment groups.

### Oncologist characteristics and practice patterns

Table 1 provides physician and practice characteristics of the final sample (*n* = 217). Approximately half of respondents were male (49%), and the mean age was 45.9 years (SD: 12.8). Twenty-nine respondents (13%) had received additional training in survivorship care. When queried about usual practice patterns related to survivorship care, oncologists reported following their patients for 5–10 years (43%) or “indefinitely” (30%) after completion of primary treatment, with only 26% transitioning care to another provider within the first 5 years. Post-treatment appointments generally consisted of patients being seen by an oncologist (62%) versus an advanced practice provider (16%), and occurred in regular oncology clinics (70%) versus breast cancer-specific (16%) or dedicated survivorship clinics (11%).

**Table 1:** Sample characteristics (*n* = 217)

Physician characteristics	Subgroup	Number	%
Mean Age (±SD), years	45.9 (±12.8)		
Mean years since medical school graduation (±SD), years	19.3 (± 12.6)		
Gender	Female	79	36.4
	Male	107	49.3
	Prefer not to say	6	2.8
	Missing	25	11.5
Race/ethnicity	Non-Hispanic White	105	48.4
	Asian	59	27.2
	Hispanic White	8	3.7
	Black	1	0.5
	Prefer not to say	19	8.8
	Missing	25	11.5
Faculty appointment in a medical school	Yes	87	40.1
	No	107	49.3
	Missing	23	10.6
Medical training in the U.S.	Yes	176	81.1
	No	18	8.3
	Missing	23	10.6
Formal training in survivorship care	Yes	29	13.4
	No	164	75.6
	Missing	24	11.1
Hours in direct patient care per week	<30	62	28.6
	>30	155	71.4
Number of patients with new cancer diagnosis per month	1–20	131	60.4
	>20	86	39.6
Number of patients with new breast cancer diagnosis per month	1–10	160	73.7
	>10	57	26.3
Practice characteristics	Subgroup	Number	%
Practice type	Academic	108	49.8
	Hospital-based	68	31.3
	Other	41	18.9
Geographic region	Midwest	36	16.6
	Northeast	56	25.8
	Southeast	54	24.9
	Southwest	23	10.6
	West	36	16.6
	Missing	12	5.5
Pre-treatment consultation duration (minutes)	<60	119	54.8
	>60	98	45.2
On-treatment appointment duration (minutes)	<30	201	92.6
	>30	16	7.4
Post-treatment appointment duration (minutes)	<15	32	15
	15–29	171	79
	30–59	13	6
	Missing	1	0.5
Routinely inform about potential acute effects	Yes	215	99.5
	No	1	0.5
	Missing	1	0.5
Routinely inform about potential long-term effects	Yes	215	99.5
	No	1	0.5
	Missing	1	0.5
Routinely inform about potential late effects	Yes	203	95.3
	No	10	4.7
	Missing	4	1.8
Usual duration of post-treatment care (years)	<5 years	57	26.3
	5–10	94	43.3
	Indefinitely	65	30.0
	Missing	1	0.5
Usual clinician for post-treatment care	Oncologist	135	62.2
	Advanced practice provider	35	16.1
	>1 clinician	43	19.8
	Other	3	1.4
	Missing	1	0.5
Usual clinical setting for post-treatment care	Regular oncology clinic	151	69.6
	Breast cancer-specific clinic	36	16.6
	Survivorship-focused clinic	23	10.6
	Other	6	2.8
	Missing	1	0.5
Routine use of consent for chemotherapy	Yes	190	87.6
	No	24	11.1
	Missing	3	1.4
Oncology care model site	Yes	50	23.0
	No	164	75.6
	Missing	3	1.4

When asked whether or not they routinely inform patients with breast cancer about potential acute (e.g., nausea, myelosuppression), long-term (e.g., peripheral neuropathy, persistent fatigue, cognitive impairment), and late (e.g., cardiotoxicity, subsequent malignancy) effects, 100%, 99.5%, and 95.7% of oncologists reported doing so, respectively. Variations in the timing of these discussions along the cancer care trajectory were noted. Most oncologists informed patients about potential long-term (80%) and late (65%) risks prior to initiating treatment, while a subset of oncologists preferred to provide information about long-term (11%) and late effects (24%) at completion of primary treatment. Formally consenting patients when starting chemotherapy was reported by 87% of the sample, and 23% described their practice setting as a demonstration site for the OCM.

### High engagement in core survivorship services

**Fig. 2:**
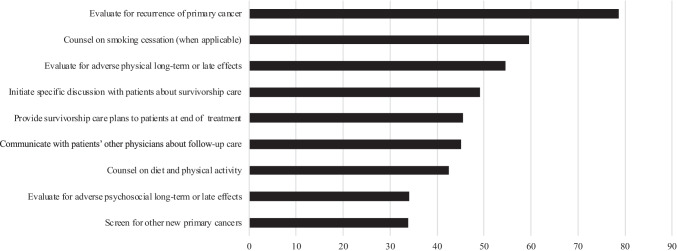
Frequency of high engagement in core survivorship services

Across the nine core services, oncologists reported high engagement, defined as “always/almost always,” most frequently in evaluation for cancer recurrence (79%) and least frequently in assessment of adverse psychosocial long-term and late effects (34%) and screening for subsequent primary cancers (34%) (Figure 2). Less than half of the sample reported high engagement in other core services, such as providing formal survivorship care plans (46%), discussing plans for survivorship care with patients (49%), communicating with patients’ other physicians (45%), and counseling on diet and physical activity (42%).

Associations between physician and practice characteristics and high engagement in the nine core activities were examined with logistic regression analyses. Longer duration of follow-up (more than 5 years after primary treatment) significantly increased the likelihood of high engagement in several services, such as assessing for both physical (OR = 4.61, 95% CI 2.27–9.74) and psychosocial (OR = 3.47, 95% CI 1.57–8.32) long-term and late effects, evaluating for recurrence (OR = 3.67, 95% CI 1.72–8.05), and discussing plans for survivorship care (OR = 1.37, 95% CI 1.37–5.57), relative to earlier transition of care (*p* < .01). Lack of survivorship training decreased the odds of high engagement in screening for other new primary cancers (OR = 0.43, 95% CI 0.19, 0.97, *p* = 0.04) and evaluating psychosocial long-term and late effects (OR = 0.35, 95% CI 0.15, 0.78, *p* = 0.01) than those who received formal training.

### Survivorship care composite score

To understand overall engagement in the various core services and delivery of comprehensive care, a survivorship care composite score was calculated for each respondent. The untransformed survivorship care composite score for the total sample ranged from 2.11 to 5.0, with a mean of 4.13, median of 4.33, and standard deviation of 0.65. Independent associations between physician and practice characteristics and the survivorship care composite scores were examined in a multivariable linear regression model with results shown in Table 2. Physician and practice characteristics explained 20.2% of the variance (*R*^2^ = 0.202, F(11,165) = 3.79, *p* < 0.001) in the model, with four variables that were statistically significant (*p* ≤ .05). A lack of formal survivorship training (*b* = −0.076, *p* = 0.036) and “not usually” informing patients about potential late effects (*b* = −0.199, *p* = 0.003) were associated with lower survivorship care composite scores. Informing patients about long-term and late effects at the time of treatment completion was associated with higher scores, but not statistically significant. Longer duration for pre-treatment consultation appointments (more than 60 minutes) was associated with a higher survivorship care composite score (*b* = 0.071, *p* = 0.006). Following breast cancer survivors after treatment completion “indefinitely” (*b* = 0.086, *p* = 0.023) was also associated with increased composite scores when compared to earlier transitions of care*.*

**Table 2:** Final regression model of survivorship care composite score

Model	Coefficient	Std. error	*p* value
Intercept	1.638	0.128	0.000
Treats >20 patients with new cancer diagnosis per month	−0.037	0.026	0.175
Pre-treatment appointment: > 60 minutes	0.073	0.026	0.006
Informs patients about long-term effects during treatment	−0.020	0.045	0.651
Informs patients about long-term effects at treatment completion	0.075	0.046	0.107
Informs patients about late effects “not usually”	−0.199	0.067	0.003
Post-treatment follow-up: 5–10 years	0.062	0.032	0.056
Post-treatment follow-up: “indefinitely”	0.086	0.037	0.023
Male oncologist	−0.053	0.027	0.052
No training in survivorship care	−0.076	0.036	0.038
Age	−0.008	0.004	0.071
Years since medical school graduation	0.008	0.004	0.068

## Discussion

In this oncologist survey, we examined current practices in breast cancer survivorship and explored factors associated with guideline-concordant care. A survivorship care composite score was created for each respondent to reflect practices across nine core survivorship activities, and used to understand patterns of care. While oncologists reported high engagement in surveillance for recurrence, other core activities were not performed as consistently. In 6 of 9 core services, less than half of oncologists reported high engagement. These patterns suggest substantial gaps in guideline-concordant care and important missed opportunities in communication, prevention, and early detection of survivorship issues.

Of particular concern, only one-third of oncologists reported that survivors in their practices consistently receive screening for subsequent primary cancers (SPCs), a leading cause of death among cancer survivors. Approximately 18% of new cancer cases are SPCs occurring in individuals with a prior history of cancer [4]. Breast cancer survivors face increased risk of SPC incidence and related mortality compared to the general population, as shown in a recent analysis of long-term cancer survivors [4]. Furthermore, many SPCs are associated with behavioral factors, such as obesity and tobacco, reinforcing the need for health promotion strategies in routine survivorship care to help mitigate this risk [21]. For these reasons, both the ACS-ASCO guideline and recent updates to the NCCN guidelines emphasize the importance of risk reduction of SPCs [8, 22].

Findings also highlight low engagement in routine assessment and management of psychosocial long-term and late effects, particularly when compared to physical effects. Psychosocial effects, such as distress, depression, fear of recurrence, and anxiety, are common, yet often go unrecognized in breast cancer survivors and are associated with poorer outcomes [23]. Routine screening and treatment for psychological distress is now considered a quality standard across the cancer care trajectory [24]. Furthermore, the ACS-ASCO guideline provides specific guidance regarding commonly experienced psychosocial issues, brief, validated tools for assessment and screening, and appropriate resources for further evaluation that can be implemented in various clinical settings.

The findings underscore challenges of achieving the comprehensive vision for survivorship care described in the guidelines in real-world practice settings. Although the ACS-ASCO guideline was originally intended for primary care providers (PCPs), this represents a major paradigm shift for oncology care and does not reflect current realities of clinical practice. Instead, our findings corroborate evidence from other studies indicating that oncologists continue to provide long-term follow-up care to most breast cancer survivors, rather than engaging in “shared care” or fully transitioning care to primary care or other clinicians. Consequently, oncologists are the central clinical professionals who deliver long-term survivorship care and are largely responsible for whether or not patients receive the services described in the guidelines [25]. In our study, long-term follow-up by oncologists was associated with significantly better composite scores, yet an oncologist-based model for long-term survivorship care is increasingly unsustainable. With an aging population, new cancer cases are expected to rise significantly and will require active management from oncology specialists [26]. The ability of oncology practices to continue seeing large numbers of breast cancer survivors will become increasingly difficult as a result. Due to the long life expectancy of breast cancer survivors, health systems will need to develop alternative strategies for effectively delivering long-term follow-up care. It is also important to note that although longer duration of follow-up was associated with better survivorship composite scores, engagement in most core services was sub-optimal, indicating the shortfalls of this oncologist-only paradigm. Therefore, innovative models of comprehensive survivorship care that employ an ongoing, team-based chronic care approach are needed, as well as a focus on the current gaps in delivery of psychosocial services to long-term survivors.

Several models for survivorship care have been proposed, but few have been rigorously tested and there is consensus that no one size can fit all [27]. Risk-stratified models and models of “shared care” between oncology and primary care providers warrant further investigation; however, numerous barriers to PCP involvement in survivorship care exist [28]. PCPs, like oncologists, face increased workloads, time constraints, and anticipated workforce shortages [27]. Concerns about PCPs’ knowledge and skills related to cancer survivorship have also been expressed by oncologists and patients, resulting in additional barriers to transitions of care [28]. In a recent survey of 127 PCPs, one-third reported no prior involvement in survivorship care, and only 16% were aware of the ACS/ASCO guideline [29]. Fragmented communication between PCPs and oncologists exacerbates these issues, particularly without integration of electronic health records across care settings [30]. In actuality, PCPs should remain involved in the care of their patients while undergoing anti-cancer treatments to enhance survivorship-related communication and planning throughout the cancer care trajectory. This type of dialogue could facilitate “shared care” from the day of diagnosis, and set the stage for ongoing shared responsibilities and clear transitions, when appropriate, across the core survivorship services, as well as incorporation of other specialists if long-term issues persist.

Our results signify modest progress in the uptake of survivorship care plans, with 45% of respondents reporting “always/almost always” using them compared to less than 10% in an older study [31]. Proactive discussions about potential long-term and late treatment effects were also important drivers of higher composite scores. Dedicated survivorship care planning visits may offer opportunities for clinicians to initiate or ideally revisit these discussions and provide anticipatory guidance about potential post-treatment issues. Lack of survivorship training was significantly associated with lower composite scores, which is consistent with prior research that demonstrated relationships between training and likelihood to provide survivorship care plans and discuss plans for cancer-related follow-up care [31]. Since only 13% of our sample reported survivorship training, and no significant improvements in training were shown in the younger ASCO participant group, additional opportunities for training and continuing education are needed to create a workforce competent in survivorship care.

### Limitations

This study has several limitations. Despite utilizing established strategies for recruitment in physician surveys, such as automated reminders, monetary incentives, and self-selected mode of administration [32], the low response rate and small sample size limit generalizability. Individuals who responded from both databases were likely motivated by personal interest in survivorship, contributing to selection bias. While low response rates in physician surveys are well-documented, this study may also reflect contemporary issues of survey fatigue, particularly using electronic strategies and heightened institutional security measures, challenges in acquiring databases with accurate contact information for physicians, as well as competition with survey requests from well-funded marketing and pharmaceutical corporations. Other limitations include the cross-sectional design and the self-reported nature of the data, which can introduce social desirability bias. However, even within this motivated sample, there was substantial variation in practice patterns, which further supported many barriers identified in our previous qualitative research [10].

## Conclusion

Improving the quality of survivorship care for the growing population of breast cancer survivors is a high priority. Disease-specific clinical guidelines are available and provide a valuable starting point, but will require extensive translational efforts to integrate into routine care. Improved understanding of current practice patterns among oncologists can help inform the development of innovative models of care and best practices regarding clinical workflow to ease the complexity of survivorship care delivery over long periods of time and across large patient populations.

## Supplementary Information

Below is the link to the electronic supplementary material.

Supplementary file1 (PDF 27 KB)

## Data Availability

Inquiries about data usage should be directed to Dr. Brauer (ebrauer@ucla.edu).

## Code availability

Inquiries about code availability should be directed to Dr. Brauer (ebrauer@ucla.edu).
