# Supplementary material for: Current practice patterns and gaps in guideline-concordant breast cancer survivorship care
Source: J Cancer Surviv. 2021 Dec 30;17(3):906–15. doi: 10.1007/s11764-021-01152-1 (PMC9243187; doi:10.1007/s11764-021-01152-1)
Supplement: Supplementary file 1 — Supplementary file1 (PDF 27 KB) [file 11764_2021_1152_MOESM1_ESM.pdf]

BREAST CANCER SURVIVORSHIP CARE

Medical Oncologists Survivorship Survey (MOSS)

[EXCERPT]

UCLA Investigators:

Patricia A. Ganz, MD

Elisa F. Long, PhD

Eden R. Brauer, PhD, RN

Conducted By:

Center for Cancer Prevention and Control Research

University of California, Los Angeles

Funded By:

Susan G. Komen Foundation

Introduction to the Survey

Thank you for agreeing to complete this survey about how you deliver survivorship care. For each question in the survey, please circle the answer which applies to you.

I: Eligibility

1) Are you a medical oncologist?
    a. Yes
    b. No

2) Do you currently provide direct care to at least some patients with breast cancer?
    a. Yes
    b. No

    If you answered "Yes" to both questions above, please continue the survey below.

    If you answered "No" to question 1 or 2, thank you so much for your interest, but you are not eligible to participate. Please stop here and return the survey to us in the postage paid return envelope.

II: Practices in Survivorship Care

1) In a typical work week, **how many hours** do you spend in direct patient care activities? (Please include all time you spend directly related to patient care, patient record keeping, patient related office work, and travel time connected with seeing patients. Please exclude time on call when not actually working.)
    a. 0-10 hours
    b. 11-20 hours
    c. 21-30 hours
    d. 31-40 hours
    e. 41-50 hours

2) On average, **how many patients** with a new diagnosis of <u>any cancer</u> do you treat in a month?
    a. 1-10 patients
    b. 11-20 patients
    c. 21-30 patients
    d. 31-40 patients
    e. 41-50 patients

3) On average, **how many patients** with a new diagnosis of <u>breast cancer</u> do you treat in a month?
    a. 1-5 patients
    b. 6-10 patients
    c. 11-20 patients
    d. 21-30 patients
    e. More than 30 patients

4) Which of the following best describes your practice?

a. Solo or up to three-person practice
b. Single specialty group
c. Multi-specialty group
d. Hospital (not including the VA)
e. VA Hospital
f. Academic practice
g. Staff/group model HMO
h. Other (please specify): _______________

5) In which state is your main clinical practice? _________________________

6) How much time do you typically schedule for the following types of visits (please indicate the average number of minutes):

Pre-treatment consultation with newly diagnosed patient	a. Fewer than 60 minutes<br>b. 60 minutes<br>c. More than 60 minutes
Routine visit with a patient currently receiving chemotherapy	a. Fewer than 15 minutes<br>b. 15-30 minutes<br>c. More than 30 minutes
Routine follow-up visit with a patient after completion of chemotherapy	a. Fewer than 15 minutes<br>b. 15-30 minutes<br>c. More than 30 minutes

Definition of Key Terms

Acute effects (“side effects during treatment”) – Side effects that occur during treatment and tend to resolve once treatment is completed [e.g., nausea and vomiting, myelosuppression, hair loss].

Long-term effects – Post-treatment effects that start during chemotherapy treatment and then persist for months to years after treatment completion. Typically patients and clinicians have difficulty understanding how and why these symptoms continue after chemotherapy treatment ends [e.g., persistent peripheral neuropathy, persistent fatigue, cognitive impairment, sexual dysfunction].

Late effects – Rare, serious problems related to cancer and its treatment that can occur months to years after treatment ends [e.g., secondary cancers, cardiotoxicity]

7) At what time point do you focus on discussing potential effects of treatment with patients who are being treated for breast cancer in your practice?

a. Acute effects	☐ Before treatment begins<br>☐ During treatment<br>☐ At treatment completion<br>☐ Not usually
b. Long-term effects	☐ Before treatment begins<br>☐ During treatment<br>☐ At treatment completion<br>☐ Not usually
c. Late effects	☐ Before treatment begins<br>☐ During treatment<br>☐ At treatment completion<br>☐ Not usually

8) Do you routinely consent patients when they are starting chemotherapy in your practice?
   a. Yes
   b. No

9) In your practice, how long do you typically follow breast cancer patients after the end of adjuvant chemotherapy treatment?
   a. <1 year
   b. 1-2 years
   c. 3-5 years
   d. 5-10 years
   e. Indefinitely

10) In your practice, who typically sees patients who have completed primary treatment at follow-up appointments?
   a. I do
   b. Advanced practice provider (NP or PA) in my practice
   c. More than one clinician (please specify): ____________________
   d. Other (please specify): __________________________________

11) In your practice, where do patients who have completed primary treatment for breast cancer go for follow-up appointments?
   a. Regular oncology clinic visit with me
   b. Breast cancer-specific clinic
   c. Survivorship-focused clinic
   d. Other (please specify): __________________________________

12) Is your practice setting a demonstration site for the Oncology Care Model (OCM), a model of care that is currently being tested by The Center for Medicare & Medicaid Services Innovation Center at sites around the country?
   a. Yes
   b. No

13) Thinking about how care is delivered to cancer survivors, how often does your practice:

a. Provide formal survivorship care plans to patients at the end of primary treatment	☐ Always/ Almost Always<br>☐ More than half the time<br>☐ About half the time<br>☐ Less than half the time<br>☐ Rarely /Never
b. Communicate with patients' other physicians about plans for follow-up care	☐ Always/ Almost Always<br>☐ More than half the time<br>☐ About half the time<br>☐ Less than half the time<br>☐ Rarely /Never
c. Initiate a specific discussion with patients regarding survivorship care and a follow-up plan	☐ Always/ Almost Always<br>☐ More than half the time<br>☐ About half the time<br>☐ Less than half the time<br>☐ Rarely /Never
d. Screen for other new primary cancers	☐ Always/ Almost Always<br>☐ More than half the time<br>☐ About half the time<br>☐ Less than half the time<br>☐ Rarely /Never
e. Evaluate patients for recurrence of primary cancer	☐ Always/ Almost Always<br>☐ More than half the time<br>☐ About half the time

	☐ Less than half the time<br>☐ Rarely /Never
f. Evaluate patients for adverse **physical** long-term or late effects	☐ Always/ Almost Always<br>☐ More than half the time<br>☐ About half the time<br>☐ Less than half the time<br>☐ Rarely /Never
g. Evaluate patients for adverse **psychosocial** long-term or late effects	☐ Always/ Almost Always<br>☐ More than half the time<br>☐ About half the time<br>☐ Less than half the time<br>☐ Rarely /Never
h. Counsel on diet and physical activity	☐ Always/ Almost Always<br>☐ More than half the time<br>☐ About half the time<br>☐ Less than half the time<br>☐ Rarely /Never
i. Counsel on smoking cessation (when applicable)	☐ Always/ Almost Always<br>☐ More than half the time<br>☐ About half the time<br>☐ Less than half the time<br>☐ Rarely /Never<br>☐

14) In what year were you born?

_ _ _ _ (year)

15) How do you describe yourself?
- ☐ Female
- ☐ Male
- ☐ Prefer not to say

16) Which best describes your race?
- ☐ White
- ☐ Black or African American
- ☐ Asian
- ☐ Pacific Islander
- ☐ American Indian
- ☐ Alaskan Native
- ☐ Other or combination: (please specify):____________
- ☐ Prefer not to say

17) Are you of Hispanic or Latino ethnicity?
- ☐ Yes
- ☐ No
- ☐ Prefer not to say

18) In what year did you graduate from medical school?

_ _ _ _ (year)

19) Did you receive your medical training in the United States?
- ☐ Yes
- ☐ No (If no, please specify): ____________

20) Do you currently hold a faculty appointment at a medical school?
- ☐ Yes
- ☐ No

21) Did you receive additional training in cancer survivorship?
- ☐ Yes
- ☐ No
